# Factors Associated with Workplace Injuries Among Shift Work Nurses: A Cross-Sectional Study in an Ecuadorian Sample

**DOI:** 10.3390/nursrep15020044

**Published:** 2025-01-27

**Authors:** Germán Prados, Ángela Mendoza-Vinces, Martha Holguín, Jacobo Cambil-Martín, Laura Fernández-Puerta

**Affiliations:** 1 Department of Nursing, School of Health Sciences, University of Granada, 18071 Granada, Spain; germanprados@ugr.es (G.P.); jcambil@ugr.es (J.C.-M.); 2 Mind, Brain and Behavior Research Center (CIMCYC), University of Granada, 18071 Granada, Spain; 3 Department of Nursing, Faculty of Health Sciences, School of Medicine, Catholic University of Santiago de Guayaquil, Avenida Carlos Julio Arosemena, km 1.5, Guayaquil 090615, Ecuador; mholguinjime@gmail.com; 4 Valencia Clinic University Hospital, 46010 Valencia, Spain; laura.fernandez@universidadeuropea.es; 5 Department of Health Sciences, European University of Valencia, 46010 Valencia, Spain

**Keywords:** shift work disorder, nurses, needlestick injuries, insomnia, sleepiness

## Abstract

**Background/Objectives:** Shift work schedules and mental and physical workloads affect the sleep homeostasis of nurses, increasing the risk of occupational injuries. This study aimed to investigate the relationship between sleep disturbances caused by shift schedules and the occurrence of needlestick and sharps injuries (NSIs) among nurses, considering significant worker and occupational factors. **Methods:** A total of 348 nurses from five hospitals of Santiago de Guayaquil, Ecuador, participated in this cross-sectional survey. Data on sociodemographic and occupational characteristics, work schedules, and NSI incidents during the previous six months were collected. Emotional status, sleepiness, and insomnia symptoms were assessed using validated questionnaires. Additionally, nurses with night shifts (fixed or rotating) were specifically assessed to estimate the relationship between NSIs and insomnia or sleepiness symptoms related to these types of shift work using logistic regression analyses. **Results:** Nurses whose schedule included night shifts showed a higher prevalence of NSIs than those with other shifts (33.2% vs. 29.0%; *p* < 0.05). High levels of depression, anxiety, and stress were associated with having had an NSI in the previous six months. Logistic regression showed that female sex (adjusted odds ratio, aOR 4.62, 95% CI: 1.65–12.97), less experience in the current clinical setting (aOR 3.12, 95% CI: 1.46–6.57), the use of psychotropic drugs (aOR 4.46, 95% CI: 1.51–13.17), and insomnia and sleepiness symptoms due to shift work (aOR 2.61, 95% CI: 1.15–5.91) increased NSI risk among nurses with night shifts. **Conclusions:** There is an acute need to explore the complex relationship between sleep troubles linked to shift work schedules, occupational factors, and the risk of occupational injuries and propose preventive strategies for enhancing nurses’ sleep health and workplace safety.

## 1. Introduction

The prevalence of having had at least one needlestick and/or sharps injury (NSI) in the previous 12 months has been estimated to range between 19.9% and 54.0% in healthcare workers (HCWs) [1,2,3,4].

NSIs pose a serious risk of blood-borne infections such as hepatitis B (HBV), hepatitis C (HCV), and HIV [5], as well as diseases due to other pathogens. According to the World Health Organization, post-exposure rates among HCWs are 39% for HCV, 37% for HBV, and 4.4% for HIV [6]. Apart from the clinical burden, NSIs are an economical burden for the healthcare system, which must cope with associated direct and indirect costs (e.g., testing for infections, post-exposure prophylaxis, HCW follow-up, and/or treatment of blood-borne viral infections) [7].

NSIs are associated with a high impact on the psychological wellbeing of healthcare workers. Fear and anxiety from worrying about the possible consequences of exposure can lead such workers to display symptoms of post-traumatic stress, anxiety, and depression [8,9,10]. Psychological distress related to NSIs can cause adverse functional changes across life domains of HCWs, deteriorating their quality of life [9,11]. Short-term consequences related to work include work absence, dissatisfaction with the current job and the wish to change to another department, and even departure from the profession [10,11,12].

The causes of NSIs are multifactorial, and the scientific literature has identified the main risk factors for this type of occupational injuries, which include professional and workplace-related factors and occupational health. Being a nurse and being a woman [2,10,13,14] are professional risk factors for having an NSI. Additionally, HCWs with longer work experience, greater knowledge of universal precautions, and training in infection prevention and safety have a lower risk of having an NSI [2,15].

Regarding workplace factors, the clinical settings in which NSIs are most frequent are the operating room/recovery room and patient room/ward, accounting for 40.4% and 26.5% of cases, respectively, according to the latest EPINet report [14]. Other workplace factors include recapping needles, using suture needles, disposing of waste, and administering injections [4,13,14]. When they are used, universal precautions and standard safety procedures are professional-based factors for not having an NSI. However, another factor is a well-established organizational safety climate promoting standard procedures, occupational health and safety systems, motivation, and safety knowledge, which in turn leads to safer behaviors and fewer occupational accidents and injuries [1,10,16,17].

Furthermore, regarding occupational health, there is evidence that mental and physical stress associated with the workload can contribute to a higher risk of NSIs in HCWs [17,18,19]. Specifically, shift work and poor sleep are two important factors that contribute to chronic fatigue in registered nurses [20,21,22]. In fact, rotating shifts and night shifts have been associated with serious impacts on natural sleep/wake rhythms. This results in sleep-related impairment characterized by shift-related insomnia and/or excessive sleepiness and difficulties for physical and mental recovery, increasing the risk of work-related accidents [20,23,24,25]. In fact, circadian rhythm sleep–wake disorder, known as shift work disorder (SWD) [26], is highly prevalent in nurses, adding a higher risk of NSIs [24,27,28].

A strong relationship has been observed between shift work organization and the risk of NSIs [20] and the high prevalence of SWD that can cause nurses to have this kind of occupational accident. Yet, to our knowledge, no studies have directly analyzed the relationship between symptoms of insomnia and/or sleepiness caused by shift schedules and NSIs in nurses. Considering this, the aim of this study was to explore this relationship in a sample of Ecuadorian nurses, including the main worker and workplace factors described by the literature that can increase the risk of having an NSI. Finally, we hypothesized that nurses with shift work-related sleep troubles would have a higher risk of NSIs.

## 2. Material and Methods

### 2.1. Study Design and Setting

We conducted a cross-sectional study among nurses employed in four public hospitals and one private hospital in the city of Santiago de Guayaquil, Ecuador. The healthcare system in Ecuador is predominantly public and provides coverage for uninsured individuals, while the private sector—accounting for only about 3% of the population—operates independently of the public healthcare services. All participants completed an anonymous online questionnaire.

### 2.2. Participants and Data Collection

Recruitment was carried out by making a formal request to conduct the research in each hospital. After the initial acceptance, a member of the research team (AMV) met with hospital directors to explain the study objectives and recruitment protocol. It was agreed with the hospital directors and nursing department heads that they would send the survey by institutional email to bedside clinical nurses in surgical and medical wards, maternal and child wards, emergency rooms, and intensive care units (ICUs) in their hospitals. Nursing students were not included in this research. A message introducing the study to potential participants and the link to the survey were provided. The following issues were included in the cover page of the online survey: general information about the study objectives, ethical guarantees and anonymization, and the recommendation not to fill out the survey if they had not worked for at least 30 days in the last three months and/or work in those three months was not in the same clinical setting. Finally, before the nurses could answer the survey, they had to give informed consent electronically. Nurses were never asked for any identifying data (e.g., name, ID number).

Data were collected from 18 to 25 October 2021 through the Google Forms application contained in the Google G Suite platform of the University of Granada (https://go.ugr.es/, accessed on 19 January 2025). Only fully completed questionnaires could be uploaded. Completing the entire questionnaire took 15 to 20 min.

The study was approved by the ethics committee of Hospital Luis Vernaza in Santiago de Guayaquil, Ecuador (Code: HLV-CEISH-2021-028).

**Figure 1:**
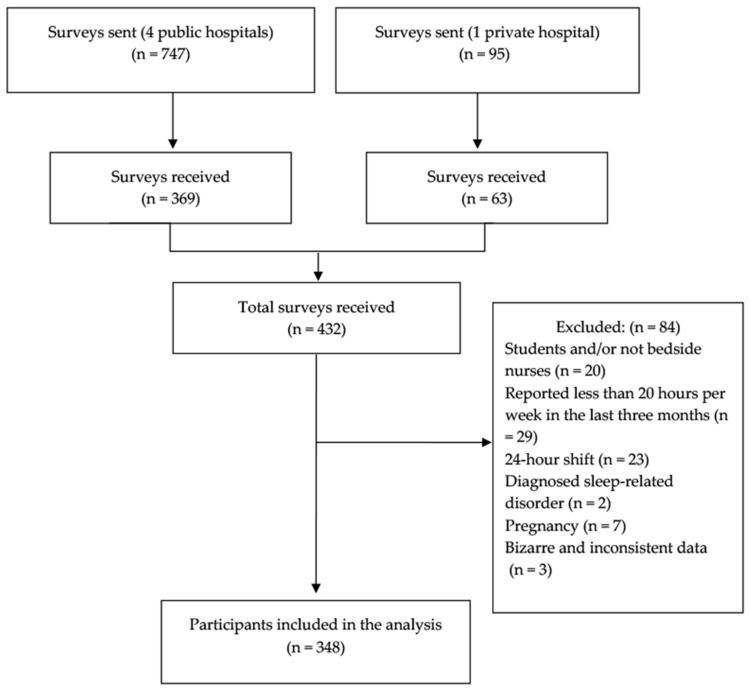
Flow diagram.

A total of 747 nurses in the public hospitals and 95 in the private one were sent the invitation to participate by email, and 369 and 63 completed the online questionnaire, respectively. Eighty-four participants were excluded by applying the following exclusion criteria sequentially: being a student and/or not being a bedside nurse (n = 20), working less than 20 h per week (n = 29), having a 24 h shift (n = 23), having a diagnosed sleep disorder (n = 2), being pregnant (n = 7), and introducing bizarre data across the whole survey (n = 3). The final study sample analyzed in this study consisted of 348 bedside clinical nurses after applying the exclusion criteria (Figure 1).

### 2.3. Demographic and Health Data

The online survey included ad hoc questions in which participants were asked about their age and sex as well as their economic and living status. We also collected data related to psychotropic drug consumption (i.e., non-benzodiazepine hypnotics, benzodiazepines, and opioids) and alcohol. According to the previous literature, psychotropic drugs can be a personal risk factor for work-related injury, so we created the binomial variable “psychotropics” to record the consumption of non-benzodiazepine hypnotics, benzodiazepines, or opioids [29].

### 2.4. Work-Related Variables

The types of clinical settings were surgical and medical wards, maternity wards, and emergency rooms or intensive care units (ER/ICUs). Responses were recoded as a binomial variable (hospital rooms vs. ER/ICUs). We asked nurses about their time of experience in the current clinical setting and a binomial question establishing a cut-off of one year or more was created. Moreover, we evaluated the types of shift schedules and working hours per week considering the last three months (less than 20 h per week was an added exclusion criterion to ensure a minimum of working days in the last three months). In the present study, nursing shift schedules were generally established as follows: all shifts lasted 8 h; day shifts went from 7.30 a.m. to 3:30 p.m., evening shifts went from 3.30 p.m. to 11.30 p.m., and night shifts went from 11.30 p.m. to 7.30 a.m. A schedule of fixed morning and/or evening shifts included five shifts per week. Fixed night shifts were not frequent among nurses in the hospitals surveyed, and those who had night shifts worked three to four nights per week. Nurses with mixed shifts rotating between night, day, and/or evening shifts worked two to three night shifts per week. Shifts of more than 10 h lasted 12 h in most cases and rarely 16 h. We did not specifically assess the features of each worker’s shift schedule after the participant answered a categorial shift-related variable in the survey considering the aforementioned information. We recoded shift work types into the following binomial variables for the statistical analyses: “8 h day shifts and shifts of more than 10 h” versus “rotating or fixed 8 h night shifts”.

Concerning workplace safety, the availability of a well-established protocol for the disposing of needles and sharp objects in the current clinical ward was screened and coded into a binomial variable (“yes” or “no”). Additionally, we asked participants if they had received training on reducing biohazards at the workplace in the last three or five years.

### 2.5. Needlestick and Sharps Injuries

We asked nurses to report NSIs they had experienced in the 6 months prior to our study. This approach was intended to minimize the time gap between the occurrence of NSIs and the self-reported assessment of workplace factors, thereby reducing the risk of recall bias associated with NSI reporting [30]. This was accomplished through two single queries: (1) “In the last six months, how many times have you had needlestick injuries in your workplace?” and (2) “In the last six months, how many times have you had cuts or lacerations caused by blades or other sharp objects in your workplace, excluding needlesticks?”. In analyzing the data, we recoded a unique binomial variable (yes/no) if the participants had given at least one affirmative answer in any of the queries surveyed; in that case, the response was scored as “yes”.

### 2.6. Self-Reported Questionnaires

A validated questionnaire, the 21-question Depression, Anxiety, and Stress Scale (DASS-21), was used. The DASS-21 consists of three seven-item subscales covering depression, anxiety, and stress, as specified in Lovibond and Lovibond [31]. The Spanish version of the DASS-21 has shown good internal consistency in a university student sample, with Cronbach’s alpha values of 0.73, 0.80, and 0.81 for the anxiety, depression, and stress subscales, respectively [32]. According to the DASS-21 interpretation manual, scores of DASS-D > 6, DASS-A > 5, or DASS-S > 9 are classified as at least moderate distress [31].

The Insomnia Severity Index (ISI) is a seven-item self-assessment tool with a five-point Likert scale, designed to measure various aspects of insomnia. Scores range from 0 to 28, with higher scores indicating greater insomnia severity. A score of 10 is recognized as indicative of insomnia complaints, despite being below the clinical threshold [33]. Morin et al. suggest that this score strikes an optimal balance between sensitivity and specificity in population studies [34]. The Spanish version of the ISI has demonstrated strong internal consistency, with scores of 0.82 in the middle-aged population [35] and 0.91 in the elderly [36].

In the Epworth Sleepiness Scale (ESS), subjects are asked to report their probability of falling asleep in eight different typical situations of daily life. ESS scores range from 0 to 24. Higher scores show higher sleepiness [37]. The ESS provides a valid measurement of sleep propensity in adults, distinguishing between individuals and diagnostic groups over the whole range of daytime sleepiness [38,39].

Having sleep-related symptoms associated with work schedules was defined using specific questions on insomnia and excessive sleepiness in relation with shifts and free time according to the scale designed by Vanttola et al. [40]. The scale was translated into Spanish following a similar process to that used by the authors with another sleep-related instrument [41]. Following Vanttola’s criteria in this questionnaire, workers who reported at least one primary symptom of SWD (e.g., insomnia and/or excessive sleepiness) “never” or “rarely” while being on holiday for at least two weeks and reported the same symptom “often” or “always” in connection to morning, evening, and/or night shifts were considered SWD cases [40]. Items in the original questionnaire that asked about insomnia and excessive sleepiness on non-workdays were not included in this study. According to scores in these questions, we classified participants into three categories: shift-related symptoms of insomnia (SW-I), shift-related symptoms of excessive sleepiness (SW-S), or both (SW-IS).

### 2.7. Statistical Analysis

Quantitative data were expressed through means and standard deviations, while qualitative data were expressed through frequencies and percentages. The sample was divided into two large groups according to the occurrence of NSIs in the past six months (i.e., none or any). Chi-square or Fisher’s exact test and Student’s t-tests were used to establish raw associations between having an NSI on one side and personal nurse characteristics, emotional status, sleep troubles (i.e., insomnia and sleepiness), and work-related variables on the other side.

Next, we selected a subsample of nurses with “rotating or fixed 8 h night shifts” to estimate the association between NSIs and having insomnia or sleepiness symptoms due to these types of shift work. Besides the crude odds ratio (cOR), we established three models: one adjusted by SW-I, a second one adjusted by SW-S, and a model adjusted by SW-IS. Three different logistic regression analyses were carried out to estimate the adjusted odds ratio (aOR) with 95% confidence intervals. In all analyses, *p* < 0.05 was treated as statistically significant. Antidepressant intake was not included in the model, as it is strongly related to depression status. Analyses were performed using the SPSS-28.0.1.0 (142) statistical package (SPSS, Inc., Chicago, IL, USA).

## 3. Results

A total sample of 348 nurses was included in the statistical analyses (see Figure 1). The prevalence of reported NSIs was 29.0% in the whole sample and 33.2% among nurses who reported having night shifts in their work schedules. The mean age was 33.64 ± 9.49 (range 22–63) and 81.3% of participants in the whole sample were female.

NSI-associated factors were explored (Table 1). Age was not a significant factor for having NSIs, but sex differences were found in this regard. Other sociodemographic factors such as the fact of cohabiting with someone else and monthly income were not determinant for having an NSI. Nurses with less than 1 year of experience in the current clinical setting had a higher percentage of NSIs (37.2%) than those with more than 1 year of experience (23.7%; *p* < 0.05).

Regarding workplace-related variables, no significant differences were found between the percentage of NSIs reported by nurses in each clinical setting (i.e., hospital rooms vs. ER/ICUs). Additionally, more than half of the whole sample (57.2%) reported having schedules including night shifts. These nurses reported a higher prevalence of NSIs compared to those that had day shifts or shifts of more than 10 h (33.2% vs. 23.5%; *p* < 0.05). Prevention training in biological hazards was not a significant factor for avoiding NSIs according to the comparative analysis. However, the availability of a disposal protocol for needlesticks and sharp objects in the clinical ward seemed to protect nurses from NSIs (53.3% vs. 25.4%, *p* < 0.001).

Regarding occupational health variables, nurses who reported having had an NSI in the last six months also reported a significantly higher use of antidepressants and psychotropic drugs compared to those who had not had work accidents (*p*’s < 0.002). Nurses in the NSI group had higher depression, anxiety, and stress scores (DASS-21) (*p* < 0.001). Additionally, these nurses had high insomnia (ISI) and sleepiness (ESS) scores (*p* ≤ 0.026).

In the subsample of 199 nurses with some night shifts, sex, antidepressant use, psychotropic drug use, experience in the current clinical setting, disposal protocol, and higher depression, anxiety, stress (DASS-21), insomnia (ISI), and sleepiness (ESS) scores were significantly associated with NSIs. This was consistent with the findings from the whole sample (see Table 2). Although weekly hours did not reach statistical significance, there was a borderline association with NSIs (*p* = 0.054). Of this subsample of nurses, 84 (42.2%) reported symptoms of having insomnia and/or sleepiness due to shift work (i.e., SW-I = 8.0%, SW-S = 7.0%; both (SW-IS) = 27.1%).

Comparative analyses testing if this subgroup of nurses was comparable to the group without night shifts in terms of demographic variables and the number of weekly hours only revealed that the former was significantly younger than the latter (32.62 ± 8.64 years old vs. 34.99 ± 10.39 years old) (*p* < 0.05).

Multivariate analyses showed that, after adjusting for covariates, the strongest predictors of NSIs were female sex, psychotropic medication use, and less than one year of experience in the current clinical setting. These factors remained consistently significant across all models (See Table 3).

Weekly hours approached significance in Models 1 (*p* = 0.062) and 2 (*p* = 0.057) and were significant in Model 3 (*p* < 0.05), suggesting a potential association with an increased risk of NSIs.

In the crude model, higher depression and anxiety scores (DASS-21) were associated with NSIs, but these associations did not remain significant after adjustment. Stress scores were not significant in either model.

Among sleep-related factors, SW-I was significantly associated with NSIs in the crude model and Model 1, while SW-S was near-significant in Model 2 (*p* = 0.057), and SW-IS was a significant predictor in Model 3. The fit of this final model was assessed using the Hosmer–Lemeshow goodness-of-fit test, which yielded a *p*-value of 0.660, indicating an acceptable fit. Additionally, the Nagelkerke R-squared value of 0.301 for Model 3 suggested that approximately 30.1% of the variance in NSI risk was explained by the variables included in the model.

## 4. Discussion

There is general evidence that shift work involving night shifts can result in sleep disturbances, reduced sleep quality, excessive sleepiness, and fatigue, which in turn may increase the risk of having work accidents. Several factors may contribute to the development of shift work disorder in nurses with work schedules that cause a misalignment of their chronobiological rhythms during long periods of their lives. Considering this, the aim of this cross-sectional study was to explore for the first time the relationship between self-reported NSIs and symptoms of insomnia and/or sleepiness caused by shift schedules in a sample of registered Ecuadorian nurses, including professional factors, workplace factors, and occupational health variables known to increase the risk of NSIs.

The prevalence of needlestick and sharps injuries (NSIs) in this study was 29.0% in the whole sample and 33.2% in nurses with night shifts in their work schedule. These findings are consistent with those of epidemiological studies in general healthcare workers [1,2,3,4,13] which have estimated the prevalence of NSIs to range between 20 and 50%. A meta-analysis focused on needlestick injuries among nurses estimated a worldwide prevalence of 40.9%; these rates were higher in developing countries compared to developed ones (59.5% vs. 30.5%, respectively) [30]. However, reviews and meta-analyses that have aimed to establish the worldwide prevalence/incidence of NSIs have included very few data from South American countries due to the small number of articles available on this region [3,4,13].

According to the demographic variables explored in the present study, we found that being female was significantly associated with a four-times-higher probability of having an NSI. These sex differences in the risk of NSIs have been reported in a recent meta-analysis that has estimated the overall incidence of NSIs among healthcare workers in World Health Organization regions. It reported a 39% incidence of NSIs in females compared to 27% in males [13]. This sex difference in prevalence may be explained by a combination of occupational, social, and psychological factors that affect job performance and increase the risk of occupational injuries among female nurses compared to their male counterparts. For example, in one study, Giorgi et al. (2018) [42] found that female nurses working in psychiatric settings often experienced poorer sleep quality than men; moreover, having long shifts as well as daytime dysfunction were significantly associated with burnout. Similarly, work–family conflict has a pronounced effect on women, including nurses, leading to stress and fatigue, which in turn elevates the likelihood of occupational accidents. Nonetheless, no studies to date have specifically examined the mediating influence of these factors on gender differences in nurses’ occupational injuries. Further research is required to elucidate these complex interactions in greater detail [43,44]. Age has also been reported as a significant risk factor for having an NSI [45]. However, in our study, this type of association was not significant. The fact that younger nurses are more likely to have the most disruptive shifts (e.g., rotating shifts) compared to older nurses in specific work contexts [46] might modulate this relationship.

Concerning other workplace variables, it has been reported that long shifts and long work hours are associated with a higher risk of having NSIs in nurses [21,45]. Our findings in the subsample of nurses with night shifts in their schedules aligned with this evidence, as weekly hours were a significant factor in the final adjusted model of the multivariate analyses. Moreover, the comparative analyses performed in the whole sample showed a significant association between having rotating and/or night shifts and having had at least an NSI in the last six months. These results agree with previous studies that found a lower incidence of NSIs when monthly night shifts were reduced [47] and fewer work-related accidents when nurses had fixed shifts [20]. Accordingly, unit and hospital policies should address the need for the adequate scheduling of the working hours of their nursing staff, as long work hours and rotating shifts can increase the risk of occupational injuries. In addition, eliminating “quick returns” (i.e., short intervals between shifts) is essential, given that they have also been associated with a higher risk of occupational accidents [20].

In our study, most nurses reported having received training in the last three years to prevent occupational biohazards, and only 12.9% of participants reported the lack of a well-established disposal protocol for needlesticks and sharp objects in their clinical setting. We found a significant association between having NSIs and not having such a disposal protocol in the total sample (Table 1) and the crude odds ratio (crude model, Table 3) in the subsample of nurses with night shifts (Table 3), but not in the adjusted model. Among all hospital procedures, disposing of waste and administering injections are the main causes of NSIs [13]. Thus, health system policies should periodically provide nurses with training on how to properly manage needles and sharp objects and dispose of waste according to evidence-based practices in this regard.

The relationship between nurses’ mental health and the risk of NSIs and/or being responsible for adverse events in patients has been described in several studies. There is evidence that the likelihood of causing these occupational accidents increases in nurses with poor overall mental health [48,49] or high levels of stress, anxiety, or depression [18,19]. In this study, comparative analyses showed a relationship between emotional status (i.e., depression, anxiety, and stress) and having had an NSI in the last six months in the total sample and the subsample of nurses with night shifts in their schedules. This statistical significance was only maintained for depression and anxiety scores in the crude model and was not reached by any emotional variable in the adjusted models of multivariate analyses. A possible explanation is that psychotropic medication use and sleep disturbances, included in the adjusted models, may mediate or influence the relationship between emotional distress and NSI risk. Very few studies on this subject have been conducted in South American countries. Yet, studies analyzing the risk of NSIs among nurses in other developing countries have shown that nurses are the HCWs most affected by mental workload because of occupational stress and the shortage of nursing resources, among other factors [50]. Additionally, the COVID-19 pandemic impacted the mental health of nurses worldwide, increasing depression, anxiety, and stress [51].

Concerning drug intake, nurses using antidepressants and psychotropics (i.e., benzodiazepines, non-benzodiazepine hypnotics, and opioids) reported a higher prevalence of occupational accidents. In addition, multivariate analyses showed that nurses who reported using psychotropic drugs were four times more likely to have an NSI. It has been reported that nurses with night shifts and/or rotating shifts take more hypnotics than daytime-only workers; moreover, the use of this medication is higher in nurses reporting SWD [52]. Our results are consistent with the evidence about these drugs, which has shown that they can hamper the performance of workers and put them at risk of occupational accidents [29,53]. In registered nurses, our findings are also in line with those of Suzuki et al. (2005), who found that hypnotic drug users had a 1.13-times-higher likelihood of having NSIs [54].

The relationship between workplace injuries and sleep-related variables underscores the critical role of sleep in occupational health and safety [55,56]. Specifically, for any type of worker, up to 13% of work injuries can be attributed to sleep problems [56]. In the present study, in the whole sample, we found significant associations between sleep-related problems (i.e., insomnia, sleepiness) and having had an NSI in the last six months using validated psychometric questionnaires (i.e., the ISI and ESS). In this context, few studies have explored the relationship between sleep-related problems and the risk of NSIs among nurses, showing contradictory results. Most of the literature analyzing predictors of NSIs includes this term together with other “medical errors” (e.g., medication errors, misidentification) [57,58,59] Thus, there is little evidence of the association between NSIs and insomnia and/or sleepiness using ad hoc questions [21,54,60]. To the best of our knowledge, only the study carried out by Demir Zencirci and Arslan [61] used a validated questionnaire to assess this phenomenon (i.e., the Pittsburgh Sleep Quality Index). That study found a positive relationship between insomnia and NSIs. However, the variability in the design of the studies, differences in nurse populations and settings, and outcome measures make it difficult to provide a comparative explanation of our findings in relation to those.

Having night and rotating shifts forces abrupt changes in the natural sleep/wake patterns of workers, resulting in a disturbance of the endogenous circadian system and its misalignment with the environment. As a result, shift workers can have acute and chronic disturbances of sleep and alertness and have an increased risk of fatigue-related occupational accidents [20,25]. Some shift workers may develop SWD, which is characterized by shift work-related insomnia and/or excessive sleepiness [26]. We used an adaptation of an SWD instrument based on the second edition of the American Academy of Sleep Medicine International Classification of Sleep Disorders [40]. The multivariate analyses showed that insomnia symptoms due to shift work in nurses with rotating and/or fixed 8 h night shifts were an independent factor for having NSIs. Nevertheless, sleepiness linked to shift work was only associated with NSIs in the crude model of the binomial regression analyses. Interestingly, when both insomnia and sleepiness symptoms were present (SW-IS), they were a significant predictor of NSIs in the final adjusted model. In the present study, a high proportion (42.2%) of nurses with night and/or rotating shifts were categorized as probably having shift-related sleep problems characterized by insomnia, sleepiness, or both (i.e., 8.0%, 7.0%, and 27.1%, respectively). Our results contrast with those of a study by Vanttola et al. in a sample of 2900 Finnish HCWs (63% were nurses). Based on the ICSD-2 and ICD-11 criteria for diagnosing SWD, the authors categorized participants according to their manifestations of shift-related primary symptoms of insomnia, excessive sleepiness, or both. They reported a total prevalence of 3.5%, 8.4%, and 6.3%, respectively [40]. Interestingly, our sample was only composed of nurses, and the instrument adaptation did not include the items in the original questionnaire asking about insomnia and excessive sleepiness on non-workdays. Moreover, we did not apply Vanttola’s exclusion criteria for employees with fewer than three non-day shifts. The SWD questionnaire requires participants to have had over two weeks of holidays to test if they meet the criteria for SWD. We did not control for this criterion. Therefore, our method of detecting sleep-related symptoms associated with work schedules cannot be equated to the diagnosis of SWD made by Vanttola. Moreover, we considered workers with fixed 8 h night and/or rotating shifts, while Vanttola and colleagues excluded permanent night workers, which leads to differences in the manner of assessing participants between studies. Despite our differences with Vanttola’s SWD prevalence rates, a recent worldwide meta-analysis conducted before the COVID-19 pandemic estimated an overall SWD prevalence of 26.5% among HCWs [62]. It showed that the prevalence of SWD among nurses ranged between 15.8 and 43.2%, with a mean of 31.65 ± 8.2. Moreover, a recent study aimed at validating a screening tool for SWD based on DSM-5 criteria for SWD found a global prevalence of SWD of 44% in a sample of nurses with shift work [63]. Thus, our estimations are likely to have reflected the situation of Ecuadorian nurses after the first wave of the pandemic, when our study took place [64,65].

This study has several limitations. First, we must mention those related to our study variables. The use of general measures of psychological status rather than instruments specifically designed to assess job-related psychosocial burden (e.g., mental workload, burnout, or secondary traumatization among nurses in the frontline during the COVID-19 pandemic) helped to better understand the occupational distress factors involved in the risk of NSIs. We did not measure chronotype, which is known to mediate the adaptation of workers to night shifts, giving them greater resilience [25]. In addition, the diagnosis of SWD requires a clinical interview and an assessment through sleep diaries or actigraphy records, which we did not use because this research was based on an online survey. The SWD questionnaire we used was a partial adaptation of the instrument developed by Vanttola and colleagues that has not been previously validated; thus, its psychometric properties remain unknown in any population.

Second, the study design also has some limitations. We considered NSIs that occurred in the last six months, so some time gaps may limit the interpretation of our results. According to our inclusion criteria, nurses had to have spent a minimum of three months in the same clinical setting to evaluate their experience in it. This may have introduced a time gap related to the occurrence of the NSI and occupational-related factors among nurses who had spent less than six months in the current workplace, as the NSI may have happened while they were still working in the previous clinical setting.

Third, the self-reported instruments used in this research asked participants to look back at the past week in the DASS-21 and at the past three months in the items on sleep-related symptoms linked to shift work schedules. NSIs might have occurred in previous periods outside the time window considered in these questionnaires, resulting in a lack of consistency between NSI occurrence and current emotional status and sleep disturbances.

Fourth, the cross-sectional design of this study makes it impossible to know if the current emotional status and sleep troubles reported by the participants were causal-related factors or variables influenced by a previous occurrence of NSIs. Additionally, data were collected from hospitals of only one city in Ecuador, and the sample size was small, which limits generalizability.

Despite the methodological limitations reported above, this research study provides important knowledge about the prevalence of needlestick and sharps injuries (NSIs) among Ecuadorian nurses in a world region where information on the prevalence of NSIs is very scarce. Moreover, this is the first research work that has explored the relationship between symptoms of insomnia and/or sleepiness caused by shift schedules and NSIs, providing evidence about the relationship between sleep problems, the use of psychotropic medication, and the risk of NSIs in nurses, a field that remains underexplored and has contradictory findings.

## 5. Conclusions

We observed a high prevalence of NSIs in bedside Ecuadorian nurses that was higher among those working night shifts. Sex differences were marked, with female nurses showing a significantly higher likelihood of experiencing NSIs compared to male nurses. Nurses with more than a year of experience in their current setting were significantly less likely to have NSIs. This study also revealed a substantial association between the incidence of NSIs and the lack of a well-established disposal protocol for needles and sharp objects. This study confirms previous evidence about mental health and the likelihood of having an NSI and reveals that the use of psychotropic drugs can increase the risk of NSIs in the workplace among nurses with night shifts. Finally, our initial hypothesis was confirmed upon observing that nurses experiencing shift work-related troubles were at higher risk of sustaining NSIs.

Our research offers important directions for future research to properly assess SWD and accurately identify nurses at risk of or experiencing SWD, develop psychometrically validated tools for screening SWD, or making clinical assessments, if necessary. There are avenues for exploring the impact of SWD on the risk of adverse events among nurses, particularly focusing on the interplay with organizational occupational variables and work-related factors that affect nurses’ mental and physical workload. By examining these connections, interventions can be tailored to address both the organizational and individual factors that compromise worker safety and mental wellbeing, ultimately fostering a safer and more supportive healthcare setting.

## Figures and Tables

**Table 1:** Characteristics of nurses.

Variables	Total (n = 348)	No NSI (n = 247)	NSI (n = 101)	*p*-Value
Age M (SD)	33.64 (9.49)	33.89 (9.30)	33.01 (9.95)	0.433
Sex n (%)				<0.001
Male	65 (18.7)	57 (23.1)	8 (7.9)	
Female	283 (81.3)	190 (76.9)	93 (92.1)	
Cohabiting n (%)				0.265
Alone	201 (57.8)	138 (55.9)	63 (62.4)	
Cohabiting	147 (42.2)	109 (44.1)	38 (37.6)	
Income n (%)				0.177
<USD 1000	121 (34.8)	93 (37.7)	28 (27.7)	
USD 1000–2000	208 (59.8)	140 (56.7)	68 (67.3)	
>USD 2000	19 (5.5)	14 (5.7)	5 (5)	
Antidepressants n (%)	18 (5.2)	7 (2.8)	11 (10.9)	0.002
Psychotropics n (%)	49 (14.1)	24 (9.7)	25 (24.8)	<0.001
Alcohol consumption n (%)				0.058
Never or occasionally	337 (96.8)	242 (98)	95 (94.1)	
Weekends	11 (3.2)	5 (2)	6 (5.9)	
Work location n (%)				0.978
ER/ICU	103 (29.6)	73 (29.6)	30 (29.7)	
General wards	245 (70.4)	174 (70.4)	71 (70.3)	
Experience in the current setting (<1 year) n (%)	137 (39.4)	86 (34.8)	51 (50.5)	0.007
Weekly hours M (SD)	40.38 (10.75)	40.19 (10.59)	40.83 (11.17)	0.614
Night shifts n (%)	199 (57.2)	133 (53.8)	66 (65.3)	0.049
No disposal protocol n (%)	45 (12.9)	21 (8.5)	24 (23.8)	<0.001
Prevention training n (%)				0.190
No, never	16 (4.6)	9 (3.6)	7 (6.9)	
Yes, more than 5 years ago	41 (11.8)	26 (10.5)	15 (14.9)	
Yes, less than 3 years ago	291 (83.6)	212 (85.8)	79 (78.2)	
DASS-Depression M (SD)	4.14 (5.13)	3.46 (4.63)	5.81 (5.88)	<0.001
DASS-Anxiety M (SD)	4.60 (5.21)	3.96 (4.86)	6.18 (5.71)	<0.001
DASS-Stress M (SD)	5.01 (5.23)	4.39 (4.96)	6.53 (5.57)	<0.001
ISI-Insomnia M (SD)	8.10 (6.03)	7.64 (6.06)	9.22 (5.83)	0.026
ESS-Sleepiness M (SD)	7.88 (5.55)	7.18 (5.21)	9.59 (5.99)	<0.001

DASS: Depression, Anxiety, and Stress Scale; ESS: Epworth Sleepiness Scale; ISI: Insomnia Severity Index; M: mean; NSI: needlestick and/or sharps injury; SD: standard deviation.

**Table 2:** Characteristics of nurses with night shifts.

Variables	Total (n = 199)	No NSI (n = 133)	NSI (n = 66)	*p*-Value
Age M (SD)	32.62 (8.64)	32.63 (8.65)	32.59 (8.69)	0.975
Sex n (%)				0.006
Male	40 (20.1)	34 (25.6)	6 (9.1)	
Female	159 (79.9)	99 (74.4)	60 (90.9)	
Cohabiting n (%)				0.644
Alone	113 (56.8)	74 (55.6)	39 (59.1)	
Cohabiting	86 (43.2)	59 (44.4)	27 (40.9)	
Income n (%)				0.075
<USD 1000	62 (31.2)	48 (36.1)	14 (21.2)	
USD 1000–2000	126 (63.3)	77 (57.9)	49 (74.2)	
>USD 2000	11 (5.5)	8 (6.0)	3 (4.5)	
Antidepressants n (%)	11 (5.5)	1 (0.8)	10 (15.2)	<0.001
Psychotropics n (%)	29 (14.6)	10 (7.5)	19 (28.8)	<0.001
Alcohol consumption n (%)				0.302
Never or occasionally	191 (96.0)	129 (97.0)	62 (93.9)	
Weekends	8 (4.0)	4 (3.0)	4 (6.1)	
Work location n (%)				0.887
ER/ICU	65 (32.7)	43 (32.3)	22 (33.3)	
General wards	134 (67.3)	90 (67.7)	44 (66.7)	
Experience in the current setting (< 1 year) n (%)	84 (42.2)	47 (35.3)	37 (56.1)	0.005
Weekly hours M (SD)	40.95 (11.94)	39.80 (12.41)	43.26 (10.66)	0.054
No disposal protocol n (%)	32 (16.1)	13 (9.8)	19 (28.8)	<0.001
Prevention training n (%)				0.504
No, never	11 (5.5)	6 (4.5)	5 (7.6)	
Yes, more than 5 years ago	168 (84.4)	115 (86.5)	53 (80.3)	
Yes, less than 3 years ago	20 (10.1)	12 (9.0)	8 (12.1)	
DASS-Depression M (SD)	4.37 (5.45)	3.43 (4.92)	6.27 (5.99)	<0.001
DASS-Anxiety M (SD)	4.68 (5.32)	3.69 (4.92)	6.67 (5.58)	<0.001
DASS-Stress M (SD)	5.20 (5.42)	4.31 (5.13)	6.98 (5.60)	<0.001
ISI-Insomnia M (SD)	8.17 (5.98)	7.57 (5.84)	9.36 (6.13)	0.023
ESS-Sleepiness M (SD)	8.07 (5.73)	7.35 (5.67)	9.52 (5.61)	0.006
Insomnia due to shift work (SW-I) n (%)	70 (35.2)	38 (28.6)	32 (48.5)	0.006
Sleepiness due to shift work (SW-S) n (%)	68 (34.2)	35 (26.3)	33 (50.0)	<0.001
Insomnia and/or sleepiness due to shift work n (%)				0.009
None	115 (57.8)	87 (65.4)	28 (42.4)	
Insomnia	16 (8.0)	11 (8.3)	5 (7.6)	
Sleepiness	14 (7.0)	8 (6.0)	6 (9.1)	
Both (SW-IS)	54 (27.1)	27 (20.3)	27 (40.9)	

DASS: Depression, Anxiety, and Stress Scale; ESS: Epworth Sleepiness Scale; ISI: Insomnia Severity Index; M: mean; NSI: needlestick and/or sharps injury; SD: standard deviation.

**Table 3:** Crude and adjusted associations between nurses working night shifts and having had an NSI in the last six months (n = 199).

Variables	Crude Model		Model 1		Model 2		Model 3	
	cOR	95% CI	aOR	95% CI	aOR	95% CI	aOR	95% CI
Age	1.00	0.97–1.03	1.00	0.96–1.04	1.00	0.96–1.04	1.00	0.96–1.04
Female sex	3.43 **	1.36–8.66	4.82 **	1.72–13.51	4.29 **	1.56–11.84	4.62 **	1.65–12.97
Psychotropics	4.97 **	2.15–11.47	4.76 **	1.61–14.07	4.28 **	1.47–12.45	4.46 **	1.51–13.17
<1 year experience	2.33 **	1.28–4.26	3.10 **	1.48–6.49	2.96 **	1.41–6.18	3.12 **	1.46–6.57
Weekly hours	1.03	1.00–1.05	1.03	1.00–1.06	1.03 *	1.00–1.06 *	1.03 *	1.00–1.06
No disposal protocol	3.73 **	1.71–8.16	1.86	0.69–4.97	1.74	0.66–4.58	1.89	0.71–5.03
DASS-Depression	2.58 **	1.37–4.85	2.72	0.76–9.70	2.59	0.73–9.20	2.70	0.77–9.55
DASS-Anxiety	2.13 *	1.16–3.93	0.69	0.20–2.35	0.67	0.20–2.27	0.64	0.19–2.16
DASS-Stress	1.97	0.99–3.92	0.42	0.13–1.35	0.44	0.14–1.42	0.41	0.13–1.36
SW-I (Insomnia)	2.35 **	1.28–4.34	2.41 *	1.14–5.07	-	-	-	-
SW-S (Sleepiness)	2.80 **	1.51–5.19	-	-	2.14	0.98–4.68	-	-
SW-IS (Both)	2.72 **	1.42–5.19	-	-	-	-	2.61 *	1.15–5.91

* *p* < 0.05; ** *p* < 0.01. Logistic regression was used. Reference categories: male, no psychotropic use, >1 year experience in the current setting, disposal protocol available. aOR: adjusted odds ratio; cOR: crude odds ratio; DASS: Depression, Anxiety, and Stress Scale; SW-I: shift-related symptoms of insomnia; SW-S: shift-related symptoms of sleepiness; SW-IS: shift-related symptoms of insomnia and sleepiness.

## Data Availability

The raw data supporting the conclusions of this article will be made available by the authors on request.

## Public Involvement Statement

No public involvement in any aspect of this research.

## Guidelines and Standards Statement

This cross-sectional study was designed and reported in accordance with the STROBE guidelines.
